# Rats’ performance in a suboptimal choice procedure implemented in a natural-foraging analogue

**DOI:** 10.1007/s10071-024-01913-2

**Published:** 2024-11-01

**Authors:** Fernanda González-Barriga, Vladimir Orduña

**Affiliations:** Facultad de Psicología, Universidad Nacional Autónoma de México, Mexico, D.F 04510 Mexico

**Keywords:** Foraging-analogue, Information, Optimality, Suboptimal choice, Rats

## Abstract

Rats and pigeons have shown striking differences in their behavior in the suboptimal choice procedure: while pigeons show a strong and consistent preference for the discriminative alternative, most studies performed with rats have found optimal preferences, and in the cases in which suboptimal preferences have been reported, those results have not been replicated. Currently, there is no consensus about the reasons for these discrepant results between species, but different explanations have been proposed either with an empirical base or exclusively in theoretical terms. In the latter category it has been proposed that the discrepancy might have arisen because of differences in the relationship between the natural foraging response of each species, and the response required in the laboratory. For analyzing this possibility, we conducted two experiments carried out within a maze that was specifically designed to allow rats to display behaviors related to their natural foraging. In experiment 1, we explored rats’ preferences when facing a discriminative alternative with probability of reinforcement (p) = 0.5, and a non-discriminative alternative with *p* = .75. In experiment 2, we evaluated preferences when the discriminative alternative had *p* = .20 and the non-discriminative had *p* = .50, rats were evaluated in a closed economy, with longer terminal links, and were allowed to escape from the outcome found. In both studies, rats showed a strong preference for the non-discriminative alternative and showed very high levels of discrimination between the positive and the negative outcomes of the discriminative alternative.

**Supplementary Information:**

The online version contains supplementary material available at 10.1007/s10071-024-01913-2.

## Introduction

The phenomenon of suboptimal choice in pigeons has generated great interest in different research areas because it seems to depart from the predictions of theories based on maximization (Fantino and Abarca 1985; Pyke et al. 1977) and because it has been regarded as a model of pathological gambling (Molet et al. 2012; Zentall 2014), and therefore, knowledge about the variables that generate it could have important clinical applications. In the prototypical suboptimal choice task, pigeons are confronted with a choice between two alternatives, a discriminative, suboptimal alternative and a non-discriminative, optimal one. The first alternative is defined by two characteristics: (a) when chosen, it presents either of two stimuli (typically with probability 0.2 and 0.8, respectively), that are reliable predictors of the outcome that will be found 10 s later; the first stimulus (positive) predicts reinforcement, while the second (negative) predicts its absence, and (b) its global probability of reinforcement (in the example, 0.2) is lower than that of the non-discriminative alternative. In contrast, the non-discriminative (ND) alternative provides either of two stimuli (ND1, ND2) that are not correlated with the outcome that will be found 10 s later, and its global probability of reinforcement is higher than that of the discriminative alternative (in the example, 0.5). Under the parameters described in the above example, it has been shown that pigeons display a strong and consistent preference for the discriminative alternative, and therefore, obtain less food than available (for reviews, see Anselme & Blaisdell, In press; McDevitt et al. 2016). Suboptimal choice requires that the subject discriminates the stimuli of the suboptimal alternative; when that discrimination is not possible because both stimuli predict the same probability of reinforcement (the suboptimal alternative is no longer discriminative), suboptimal choice disappears, i.e., subjects prefer the alternative with the higher probability of reinforcement (López-Tolsa and Orduña 2021; McDevitt et al. 2019; Stagner and Zentall 2010).

Despite the reliability of suboptimal choice in birds, the generality across species has been questioned, as several studies with rats and human participants have found optimal behavior under the same set of parameters than those employed in the demonstration of pigeons’ suboptimal behavior (Alba et al. 2021; Bodily and Bodily 2024; López et al. 2018; McDevitt et al. 2019). Much research has explored different potential variables that could explain the contradictory findings in pigeons and rats. Among them, the difference in the incentive salience of the stimuli that are employed as discriminative stimuli has been regarded as the relevant variable. Briefly, whereas studies with pigeons employ illuminated keys as stimuli, which have high incentive salience demonstrated by the strong sign-tracking behavior that they generate (Brown and Jenkins 1968), the first studies with rats employed small lights over the levers, which do not generate sign-tracking in rats and therefore are assumed to lack incentive salience. Following this argument, Chow et al. (2017) employed the presence of a lever as the positive discriminative stimulus, a blackout as the negative discriminative stimulus, and the presence of another lever as the non-discriminative stimulus, finding a preference for the discriminative alternative, which was explained by the higher incentive salience of the lever. This result seemed to solve the problem of the inconsistency between rats and pigeons in their preference in the suboptimal choice procedure; however, it has not been possible to replicate that preference in several studies that also have employed levers as discriminative stimuli and that have demonstrated appropriate levels of discrimination between the positive and the negative stimuli (Alba et al. 2018, 2021; López et al. 2018; Martínez et al. 2017; Orduña and Alba 2019).

Another explanation of the differences between pigeons and rats is based on the temporal information conveyed by the discriminative stimuli. Although the afore-mentioned difference was obtained with the same set of parameters, i.e., a single response in the initial links, and 10-s long terminal links (TL), it is possible that pigeons and rats are differentially sensitive to the TLs’ length. Following this argument, embedded in the temporal-information theoretic model (Cunningham & Shahan, 2018), Cunningham and Shahan (2019) manipulated the length of the TLs, finding that rats’ suboptimal preferences emerged when TLs were at least 30-s long. The strong preference found in this study, however, was not replicated in a later study by the same laboratory (Cunningham and Shahan 2020), as in different groups of rats tested with initial links fixed-ratio 1, fixed-interval 5 s or fixed-interval 10 s (all of them with 50-s-long TLs), no preference for the discriminative alternative was found. Furthermore, it has been shown that rats display optimal preferences with 50-s-long TLs (Alba et al. 2021). These results together suggest that the manipulation of TLs length does not have a consistent impact on suboptimal choice preferences by rats.

In a further attempt to explain the species difference, Zentall et al. (2019b) have proposed that it could have arisen because of the dissimilarities between studies with rats and pigeons in the relationship between the stimuli employed in the laboratory and those present in the natural environment of each species. More concretely, in differences in the way that those stimuli activate the search modes of the feeding-behavior subsystem (Timberlake 1993). Zentall et al. (2019b) argue that in studies with pigeons, key-pecking directly activates the focal-search mode, while in studies with rats, the search-mode activated differs according to the particular kind of stimuli employed: in those studies that have used lights or tones, a general-search mode has been activated (predictive of optimal behavior), while in those that have employed levers, the focal-search mode has been activated (predictive of suboptimal behavior). As was discussed above, several studies have found optimal behavior with procedures that employ levers as discriminative stimuli, so that it seems warranted to approach Zentall et al’s account (2019b) by testing the impact of other kind of stimuli hypothesized to be strongly-related to the activation of the focal-search mode in the natural habitat of rats.

As stated by Timberlake (1993), the focal-search mode is activated by the presentation of cues that are good predictors of the time or place of food presentation, and such mode is characterized by behavior getting more focused on food procurement. This argument, joined to the easiness with which rats perform locomotion for efficiently traversing different types of mazes (Timberlake 2002) suggests that some of the stimuli present in mazes activate the focal-search mode. This opens the possibility of studying the preferences of rats in a suboptimal choice procedure that incorporates those kinds of ecologically-valid stimuli and responses: locomotion, and spatially-defined alternatives. In the present report, we evaluated rats in a relatively-complex maze that incorporates both responses and stimuli related to the natural foraging, with the objective of analyzing its impact on preferences in the suboptimal choice procedure.

## Experiment 1

### Method

#### Subjects

Subjects were 8 Wistar rats, male, experimentally naïve, approximately four-months old, acquired from the vivarium of the Institute of Cell Physiology, Universidad Nacional Autónoma de México. Subjects were housed in pairs with free access to food and water. After habituation to the conditions of the vivarium, the food-restriction regime was implemented, which consisted in providing each subject between 12 and 15 gr. of food after the experimental session, in order to maintain them at approximately 85% of their free-feeding weight. The experiment followed the official Mexican norm NOM-062-ZOO-1999 “Technical Specification for Production, Use and Care of Laboratory Animals”.

#### Apparatus

**Fig. 1:**
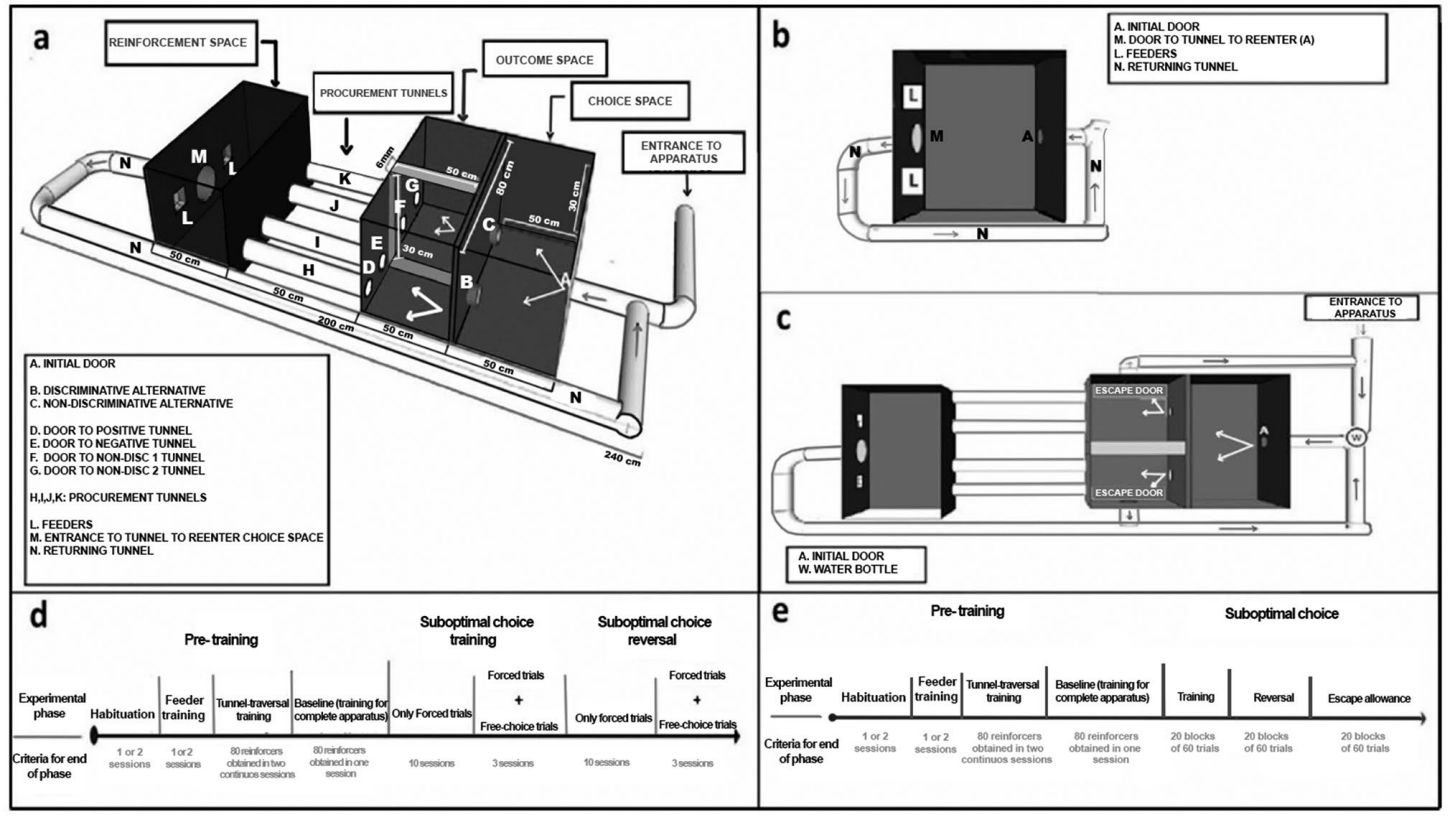
Diagram of the apparatuses employed in the different phases of the experiments. Panel a shows the apparatus used in the main conditions of experiments 1 and 2. Letters A, B, C, D, E, F, G, and M signal the location of different doors, letters H, I, J, K and N indicate the location of tunnels, and L represents the location of feeders. Panel b shows the simplified apparatus that was used for pre-training; letters have the same meaning than in previous panel. Panel c shows the location of the water bottle that was used in all phases of experiment 2, and the two new “escape doors” that were used in the “escape allowance” condition of that experiment. Panels d and e show timeline diagrams for experiment 1 and 2, respectively. The different pre-training and training phases are arranged according to the time of their presentation, and the criteria for ending each phase is indicated. See text for details

Figure 1, panel a describes the apparatus employed for the conduction of the experimental sessions, which was specifically designed for the present experiments. The apparatus consists of three inter-connected spaces that could be accessed by crossing automatic guillotine doors (MED Associates, INC., Model ENV-013ADB2) whose arch began two cm above the floor, and was 9.32-cm high. Doors were outfitted with an infrared detector (MED Associates, INC., Model ENV-254-CB), which allowed to detect crosses to the door. The infrared detector was mounted in the arch of the door, 10 cm above the floor. Each of the three spaces of the apparatus was 80-cm wide, 50-cm long and 30-cm tall; the floors and walls were made with PVC laminates 0.6-cm wide. The first space was regarded as the choice space. In the back wall of it, a door was mounted in the center (A in Fig. 1 panel a). This door had the purpose of allowing the entrance to the choice space from a PVC tube mounted in the other side of this wall. In the front wall of the choice space two additional doors (B and C in Fig. 1 panel a), were mounted 15 cm from its respective sidewall. Crossing doors B and C gave access to the second space (regarded as the outcomes space), where the different outcomes could be presented, signaled by the opening of its respective door (D, E, F, and G in Fig. 1 panel a). The outcomes space was divided in two by a PVC wall, and as a result, door B could give access only to the outcome space where doors D and E were located, while door C could give access only to the outcome space where doors F and G were located. Crossing the doors D, E, F, and G, gave access to 10-cm diameter, 50-cm long PVC tubes (tunnels H, I, J, K in Fig. 1 panel a), after which the reinforcement space (or goal box) was located. In the front wall of this space, two 5.2-cm x 5.2-cm pellet receptacles were mounted (MED Associates, Inc., Model ENV-200R2M) 7 cm above the floor and 14 cm from its respective sidewall. The pellet receptacles could be illuminated by a LED mounted in its ceiling (Med Associates, Inc., Model ENV-200RL-LED). Periodically, the pellet receptacles could receive, according to the experimental schedule, one 45-mg food pellet (Bio-Serv, Product F0165) from pellet dispensers (MED Associates, Inc., Model ENV-203 M) that were mounted in the back of the front wall. In the center of the front wall, an additional door was mounted (M in Fig. 1 panel a), which gave access to a tunnel 320-cm long (N in Fig. 1 panel a), which returned the subject to the choice space of the apparatus. Additional infrared detectors were mounted at the exit of tunnels H, I, J, K, and in the returning tube (N), at 30 cm of distance from door M. The presentation of stimuli and the collection of data were controlled by personal computers using the Medstate programming language (Med-PC-V, MED Associates, Inc.).

Figure 1 panel b, describes the apparatus employed for pre-training, which was a simplified version of that described above, and consisted only in the reinforcement space of the complete apparatus. The front wall had the same components, two pellet receptacles (L) with pellet dispensers mounted in the back of the wall, and the door M, which gave access to the return tunnel (N) that led subjects to the entrance of the apparatus (A), located in the back wall of the reinforcement space.

#### Procedure

##### Habituation and feeder training

Subjects were habituated to the apparatus described in Fig. 1 panel b over one or two 30-min-long sessions. Twenty-five pellets were placed in each of the pellet receptacles and 25 more pellets were scattered over the floor. Habituation sessions were terminated when subjects ate all the pellets during the session. During the next session, a pellet was delivered in a randomly-chosen pellet receptacle according to a variable-time 30 s (range 1.55 –90.07 s) schedule until 80 pellets had been delivered. When a pellet was delivered, the light of the pellet receptacle was turned on for 3 s. Both doors (M and A) remained closed for the entire sessions. This pre-training continued until subjects retrieved the pellet in at least 80% of the trials before the next pellet was delivered. No more than two sessions were needed to attain this criterion.

##### Training for tunnel traversal and habituation to complete apparatus

The subject was directly placed into the experimental space (see Fig. 1 panel b) and 60 s after, a pellet was delivered in a randomly-chosen pellet receptacle. Three s after that pellet was retrieved, door M was opened. When subject broke the infrared light that was located in tunnel N, 30 cm after door M, this was closed at the same time that door A was opened. When subject crossed door A, a pellet was delivered in a randomly-chosen pellet receptacle, door A was closed, and the cycle continued until 80 pellets were delivered or 40 min elapsed. This training continued until subjects obtained 80 reinforcers in two consecutive sessions.

The next day, subjects were exposed to the complete apparatus (see Fig. 1 panel a). Subjects were placed in the PVC tube that connected to door A at approximately 40 cm of distance from it. When subject crossed door A, door B or C were opened with probability 0.5, and when subject crossed it, either of its two associated outcomes were presented with probability 0.5, which was signaled by its door being open (D or E after crossing door B; F or G after crossing door C). After traversing tunnels H, I, J or K, a pellet was delivered in the pellet receptacle located in the same side of the outcome presented. Five s after pellet delivery, door M was opened, and the cycle continued for 80 trials or 60 min. When subjects obtained 80 reinforcers in this condition, the pre-training finished, and the experiment began the next session. If in this stage a subject showed a preference for either door B or door C, the discriminative alternative was assigned to its preferred door during the next stage. For subjects with no preference, the assignment was counterbalanced.

##### Suboptimal choice procedure, training

In the first ten sessions, only forced-exposure trials were presented. Sixty-four trials were presented in each session, divided in eight blocks of eight trials. In each block, four trials were assigned to each alternative. From the four forced trials for the discriminative alternative, two were assigned to the positive outcome and two to the negative outcome. From the four forced trials for the non-discriminative alternative, two were assigned to the non-discriminative (ND)1 outcome, and the other two to the ND2 outcome. Each trial began when subjects crossed door A (see Fig. 1, panel A). In that moment, either door B (discriminative alternative), or door C (non-discriminative alternative) were opened with probability 0.5. In trials of the discriminative alternative, when subject crossed door B, door A was closed and either door D or E was opened with probability 0.5. When subjects crossed the open door, door B was closed. In trials in which door D was open, after traversing tunnel H, a pellet was delivered with *p* = 1.0 and door D was closed; this tunnel was regarded as the positive outcome. In trials in which door E was open, after traversing tunnel I, no reinforcement was delivered and door E was closed; this tunnel was regarded as the negative outcome. In trials of the non-discriminative alternative, when subjects crossed door C, either door F or door G was opened with probability 0.5 at the same time that door A was closed. After traversing tunnel J or K, reinforcement was provided with probability 0.75 and the outcome door (F or G) was closed. These tunnels were regarded as the non-discriminative outcomes. Five s after reinforcement delivery or 8 s after arriving the goal box in cases in which no reinforcement was delivered, door M was opened, and when subjects traversed 30 cm of the return tunnel (N), door M was closed at the same time that door A was opened, allowing subjects to begin a new trial by traversing the return tunnel. In sessions 11, 12 and 13, two free-choice trials were added to each block of trials, for a total of 16 free-choice trials per session. In these trials, when subjects crossed door A, both door B and door C were opened; crossing either of them began the same cycle of contingencies than in forced trials, at the same time that the non-chosen door was closed.

##### Suboptimal choice procedure, Reversal

During the next 13 sessions, a change of position of the discriminative and non-discriminative alternatives was performed in order to detect any potential position bias during the training phase described above. From sessions 14 to 23, only forced trials were presented, while in sessions 24, 25 and 26, free-choice trials were added. Figure 1, panel d summarizes the different phases employed during pre-training and training and the criteria for ending each of them.

#### Data analysis

As indexes of preference, we analyzed two types of variables: (A) In forced trials, we analyzed the latencies to cross the door of both the discriminative and the non-discriminative alternatives; these latencies were defined as the time that elapsed since door B or C was opened, until subject crossed that door. For each session, the median for each subject was calculated and data from each of the last five sessions were submitted to a repeated-measures ANOVA with alternative (discriminative, non-discriminative) and sessions as within-subjects factors. (B) for sessions with free-choice trials, besides performance during forced trials we analyzed the proportion of choice for the discriminative alternative. The individual means of the three sessions of each phase (training, reversal) were analyzed with t-tests of one sample, that evaluated difference against a reference value of 0.5, which denotes indifference. As indexes of discrimination, we analyzed two types of variables: (C) the latencies to cross the door associated with each type of outcome (positive, negative, ND1 and ND2), and (D) the traversing time of each type of outcome, defined by the time that it took a subject to traverse the tunnel from its associated door to the end of the tunnel. In both cases, we calculated the individual median latencies for each outcome in each session, and data from the last 5 sessions were submitted to repeated-measures ANOVA with type of outcome and sessions as within-subjects factors. If the assumption of sphericity was violated, Greenhouse-Geisser corrections were implemented. Training and reversal data were analyzed separately.

### Results

**Fig. 2:**
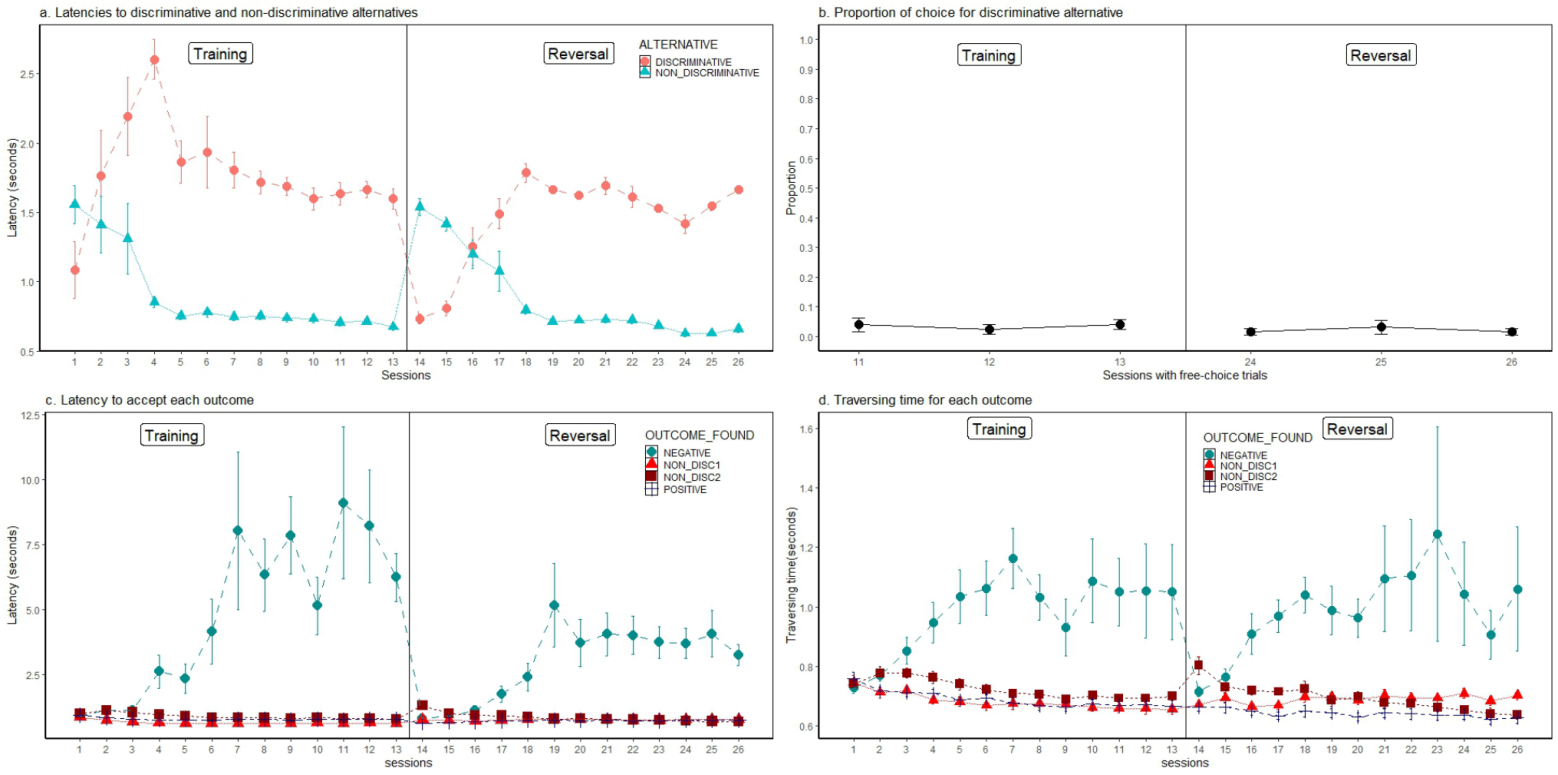
Experiment 1. Group means and standard errors (Mean ± SEM) for the different variables during each session of experiment 1. Panel a shows the latencies for entering the doors associated with the discriminative and non-discriminative alternatives. Panel b shows the proportion of choice for the discriminative alternative. Panel c shows the latency to enter the door associated with each of the four possible outcomes (positive, negative, ND1 and ND2). Panel d shows the traversing time of the tunnel associated with each of the possible outcomes. The vertical line in each panel divides data obtained during the original training condition from those obtained during the reversal phase

Figure 2 shows the group average of the main variables obtained during both forced trials and free-choice trials across all sessions of the experiment (individual data can be observed in Online Resource 4). In panel a we observe the group mean ± standard error of the mean (SEM) of the individual median latencies to enter the discriminative and the non-discriminative alternatives during forced trials. It can be observed that beginning in session 3, a shorter latency was observed for entering the non-discriminative alternative than the discriminative alternative. This difference prevailed during the remaining sessions of training. During the last five sessions, the mean latency to enter the non-discriminative alternative was 0.72 s ± 0.024 s (mean ± SEM), while the mean latency to enter the discriminative alternative was 1.64 s ± 0.065 s. An ANOVA on data from these sessions indicated significant difference between alternatives (F(1, 7) = 181.81, *p* < .0001; partial η^2^ = 0.962), and non-significant effects of sessions (F(4, 28) = 2.64, *p* = .055; partial η^2^ = 0.27) and of its interaction with alternative (F(4, 28) = 0.964, *p* = .44; partial η^2^ = 0.12). Consistent with these data obtained from forced trials, during free-choice trials (sessions 11,12 and 13, see Fig. 2, panel b) subjects showed a strong preference for the non-discriminative alternative, as the mean choice proportion of the discriminative alternative was 0.033 ± 0.014. A t-test of one sample against the reference value of 0.5 reported that the preference was significantly below random performance (t (7) = -31.71, *p* < .001, SE Cohen’s d = 2.82). In addition to these data related to preference, Fig. 2 panels c and d show data related to the discrimination between the different outcomes associated with the discriminative and the non-discriminative alternatives. Panel c shows the latency to enter the doors that indicated the positive and negative outcomes associated with the discriminative alternative, as well as the latency to enter the outcome doors associated with the non-discriminative alternative. An ANOVA indicated that the type of outcome (ND1, ND2, positive and negative) exerted a significant effect on the latency to enter its associated door (F(1, 7) = 28.68, *p* < .001, partial η^2^ = 0.80; Greenhouse-Geisser correction was implemented because of violation of the assumption of sphericity). Sessions and its interaction with type of outcome were non-significant (F(4, 28) = 0.99, *p* = .43, partial η^2^ = 0.12; and F(12, 84) = 1.01, *p* = .45, partial η^2^ = 0.12, respectively). Scheffé post-hoc analysis indicated that there was significant difference between the positive and the negative outcomes, but there was not significant difference between ND1 and ND2 outcomes. Finally, Fig. 2 panel d shows the group mean of the individual medians of the traversing times from the outcome door to the goal box for each type of outcome. It can be observed that the traversing time for the negative outcome was higher than for the other outcomes: positive, ND1 and ND2. An ANOVA evaluated the effect of type of outcome on the traversing times during the final 5 sessions. A significant effect of type of outcome was found (F(1.02, 7.13) = 10.32, *p* < .001; partial η^2^ = 0.60), but non-significant effects of sessions (F(4, 28) = 0.53, *p* = .71; partial η^2^ = 0.07) nor of its interaction with type of outcome (F(12, 84) = 0.45, *p* = .94; partial η^2^ = 0.06). Scheffé post-hoc analyses indicated that there was a difference in the traversing time of the positive and the negative outcomes, but there was not a difference in the traversing times of the ND1 and the ND2 outcomes. The analysis of these variables was also performed for data obtained during the last 5 days of the reversal phase. The results of the training phase were replicated, with two exceptions: (a) for the analysis of traversing times the factor outcome was not significant when the Greenhouse-Geisser correction was applied (*p* = .07), in contrast to data from training condition and (b) the ANOVA regarding the analysis of latencies for the discriminative and non-discriminative alternatives indicated that both the factor session and its interaction with alternative were significant, in contrast to the results obtained during the training phase in which both were non-significant. According to Scheffé post-hoc analyses of the interaction, its statistical significance arose because for the discriminative alternative, the latency during session 24 was significantly shorter than those obtained during sessions 22 and 26; for the non-discriminative alternative, in contrast, no differences between sessions were obtained. Statistical details regarding the reversal phase are provided in Online Resource 1.

### Discussion

The main objective of the present experiment was to evaluate with a novel procedure rats’ preference between a discriminative alternative with lower probability of reinforcement and a non-discriminative alternative with higher probability of reinforcement. When rats have been evaluated in the suboptimal choice procedure, invariably operant chambers have been employed either with lever presses or nosepokes as the choice response, and most of the studies have used stimuli from the visual or auditory modality; in addition, in those studies the discriminative and non-discriminative stimuli signal a fixed-time or a fixed-interval schedule of reinforcement, implying that no effort by the subject, or a minimum one is necessary to obtain the consequences. It can be argued that in rats’ natural environment, the exploitation of the best food sources is facilitated by learning their physical locations (Benedix 1993), and that the effort involved in locomotion for reaching them implies higher costs than simply waiting for the same amount of time (for a related discussion, see Aparicio and Baum 1997), which could raise the sensitivity to the discriminative properties of those stimuli. Taking this into account, we developed a foraging analogue in which the different alternatives were defined exclusively by its physical location, and locomotion towards them was the choice response. In addition, when the different outcomes were found, subjects “pursued” them with actual locomotion through a tunnel. While it can be discussed whether these characteristics are enough for the procedure to be regarded as a natural-foraging analogue, it undoubtedly allows for the evaluation of the generality of rats’ preferences in the “suboptimal choice procedure”, in a more ecologically-valid context in which the discriminative and non-discriminative stimuli are defined exclusively by its spatial location, and high effort is required for reaching its associated outcomes. Under these circumstances, we found two main results. Rats preferred the non-discriminative alternative, a finding that is supported by the analysis of two different types of variables: (a) during forced trials, the latencies for entering the non-discriminative alternative were notably shorter than those for entering the discriminative alternative, and (b) during free-choice trials the proportion of choice for the discriminative alternative was close to zero. For the analysis of the relevance of these results, it is crucial to evaluate whether rats adequately discriminated between the positive and negative stimuli of the discriminative alternative, since when pigeons cannot perform such discrimination, they prefer the optimal alternative (López-Tolsa and Orduña 2021; Stagner and Zentall 2010). In this context, the second main result -described next- validates the first one. Rats readily discriminated between the positive and the negative outcomes, as indicated by the analysis of two variables: the latency to enter the door associated with the negative outcome was larger than the latency to enter the positive-outcome’s door, and the traversing time of the tunnel associated with the negative tunnel was longer than the time for traversing the positive tunnel.

Although rats’ optimality in the “suboptimal choice procedure” has been frequently found (for a review, see Alba et al. 2021), a remarkable aspect of the present results is the small number of trials needed for observing both a preference for the optimal, non-discriminative alternative and high levels of discrimination. The fast acquisition of preference and discrimination was especially notable during the reversal phase. Related to this, another aspect of the present results is that the preference for the non-discriminative alternative was stronger than in other studies performed in operant chambers that have employed the same probabilities of reinforcement (Alba et al. 2018; Martínez et al. 2017), which have found that the final proportion of choice for the discriminative alternative after 40 sessions was between 0.20 and 0.30 both during the training phase and during reversal. Further research is needed to evaluate which aspects of the present experiments are responsible for this effect, but it is possible that evaluating subjects in conditions more similar to those present in their natural foraging contributed to it.

The procedure employed allows to perform different manipulations that have been proposed as relevant for understanding rats’ preferences in the suboptimal choice procedure. For example, it is warranted to assess the generality of the previous findings when other probabilities of reinforcement are employed. Besides it, given that it has been proposed that suboptimal preferences develop when the terminal links are lengthened (Cunningham and Shahan 2019) it is warranted to extend the outcome-tunnels’ length, which in the present procedure represents the terminal-links. In the next experiment, new probabilities of reinforcement were associated with the discriminative and non-discriminative alternatives (0.2 for the former, 0.5 for the latter), longer outcome-tunnels were presented than in experiment 1 (1.5 m instead of 0.5 m), and in order to more-closely simulate natural foraging, we performed two additional manipulations: (a) a closed economy regime was employed during the entire experiment (Hursh 1980), and (b) in a third condition, animals were allowed to escape from the outcome presented and initiate a new trial (equivalent to beginning a new food-search episode in the natural environment), a characteristic of natural environments that to our knowledge has not been simulated in studies of suboptimal choice with rats.

## Experiment 2

### Method

#### Subjects

Subjects were 6 Wistar rats, male, experimentally naïve, approximately four-months old, acquired from the vivarium of the Institute of Cell Physiology, Universidad Nacional Autónoma de México. Subjects were housed individually with free access to food and water. After habituation to the conditions of the vivarium, the food-restriction regime was implemented, which consisted in providing each subject between 12 and 15 gr. of food after the experimental session, in order to maintain them at approximately 85% of their free-feeding weight. This regime was maintained during pre-training. During the main procedure, no food was provided outside the experimental session, which had a duration of 18 h and began at 18:00. The food obtained during the sessions was enough for maintaining the weight of subjects between 80 and 92% of their free-feeding weight. The experiment followed the official Mexican norm NOM-062-ZOO-1999 “Technical Specification for Production, Use and Care of Laboratory Animals”.

#### Apparatus

The apparatus was the same than that described for experiment 1, with the following exceptions: (1) The outcome tunnels were lengthened from 50 cm to 150 cm, (2) Two escape-doors were mounted in the sidewalls of the outcomes-space, one for the outcomes associated with the discriminative alternative, and other for the outcomes associated with the non-discriminative alternative. Figure 1 panel c shows a diagram of the apparatus with the added escape doors. Infrared detectors were mounted in the return tunnels, 25 cm far from the escape door. These infrared-detectors had the objective that when they were crossed, the door of the outcome that was presented was closed and the initial door (A) was opened. Water was freely available during the entire session via a bottle that was mounted in the return tunnel, 20 cm before door A.

#### Procedure

##### Pre-training

Pre-training was identical than for experiment 1.

##### Suboptimal choice, training

The procedure was similar than in previous experiment, with the following exceptions: (1) free-choice trials were presented since the beginning of training and during the entire experiment. (2) two 45-mg pellets were delivered as reinforcement, instead of one in experiment 1, and (3) trials were presented in blocks of twelve trials, from which five were assigned to the discriminative alternative, five to the non-discriminative alternative, and two were free-choice trials. From the five trials assigned to the discriminative alternative, one presented the positive tunnel, and four the negative one. For consistency, from the five trials with the non-discriminative alternative, one presented the ND1 outcome, and four the ND2; the probability of reinforcement for ND1 and ND2 was 0.5. These modifications had as consequence that the probability of reinforcement for the discriminative alternative was 0.2, while the probability of reinforcement of the non-discriminative alternative was 0.5. This condition ended when subjects completed 20 blocks of 60 trials.

##### Suboptimal choice, reversal

A reversal phase was implemented in order to detect any potential position bias in the procedure described above. Its duration was the same than training condition, i.e., 20 blocks of 60 trials.

##### Suboptimal choice with escape allowance

Immediately after the last session of reversal, the “Suboptimal choice with escape allowance” phase was initiated. In this phase, at the same time that a particular outcome’s door was presented, its associated escape door was open. If subject took the escape route, as soon as it reached door A, a new trial began. Otherwise, the contingencies were the same than described before. The escape route was available as soon as the outcome’s door was opened, and remained available until the procurement-tunnel was completely traversed. Important to highlight is that escape was available for each of the four possible types of outcomes. This condition was maintained for 20 blocks of 60 trials. Online Resource 3 presents a brief video showing an example of a trial with escape from the negative outcome, a trial with no escape from the ND2 outcome, and a trial with no escape from the positive outcome. Figure 1, panel e summarizes the different phases employed during pre-training and training and the criteria for ending each of them.

#### Data analysis

The variables related to preference (latencies to alternatives, and proportion of choice for the discriminative alternative) were analyzed in the same way than in previous experiment, with the exception that the ANOVAS included data from the last 5 blocks of 60 trials, instead of the last five sessions. The index of discrimination was different among conditions: during training and reversal, the same indexes than in experiment 1 were analyzed, i.e., latencies and traversing times. During the condition with “escape allowance”, we analyzed as index of discrimination the proportion of trials with escape for each of the four possible outcomes, because the high proportion of trials with escape when the negative outcome was found precluded the analysis of latencies to outcomes and traversing times.

### Results

**Fig. 3:**
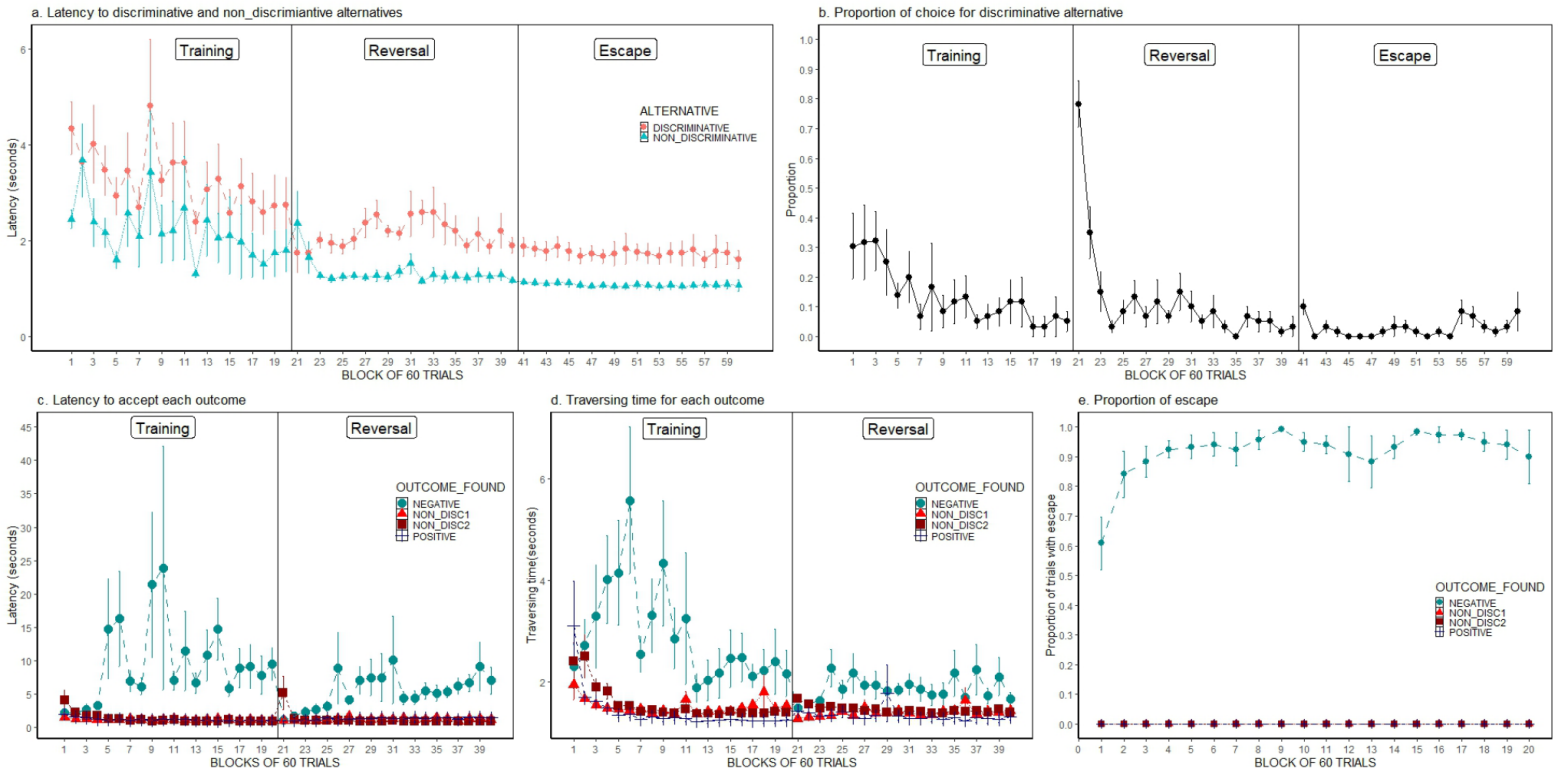
Experiment 2. Group means and standard errors (Mean ± SEM) for the different variables across blocks of 60 trials. Panel a shows the latencies for entering the doors associated with the discriminative and non-discriminative alternatives. Panel b shows the proportion of choice for the discriminative alternative. Panel c shows the latency to enter the door associated with each of the four possible outcomes. Panel d shows the traversing time of the tunnel associated with each of the possible outcomes. Panel e shows the proportion of trials with escape for each of the four possible outcomes found. Vertical lines in each panel separate data obtained during the original training condition from those obtained during the reversal phase and escape conditions (where appropriate)

Figure 3, panels a-e show the group average of the main variables obtained during experiment 2 (individual data can be observed in Online Resource 5). Panel a shows the median latency to enter the non-discriminative and discriminative doors during forced trials across all blocks of trials of the training, reversal and escape conditions. Data are grouped in blocks of 60 trials, of which 10 were free-choice trials, and 50 were forced trials (25 for each alternative, discriminative and non-discriminative). From the 25 forced trials assigned to the discriminative alternative, 5 presented the positive outcome and 20 the negative outcome; from the 25 forced trials assigned to the non-discriminative alternative, 5 presented the ND1 outcome, and 20 the ND2 outcome. As can be seen in the figure, for roughly all blocks since the beginning of training and until the 20th block, the latency to enter the non-discriminative door was shorter than to enter the discriminative door. An ANOVA performed on data from the final five blocks indicated that latencies were significantly shorter (F(1, 5) = 35.67, *p* < .01; partial η^2^ = 0.88) for the non-discriminative door (1.73 s ± 0.49 s) than for the discriminative door (2.79 s ± 0.56 s). The factor block of trials (F(4, 20) = 2.13, *p* = .11; partial η^2^ = 0.30) and its interaction with alternative (F(1.52, 7.61) = 0.14, *p* = .96; partial η^2^ = 0.03) were not significant. Consistent with the longer latencies for the discriminative alternative during forced trials, the proportion of choice for the discriminative alternative (see Fig. 3, panel b) during free-choice trials of the training condition was considerably small (0.06 ± 0.05). A t-test of one sample indicated that the proportion of choice for the discriminative alternative was significantly below random performance. (t (5) = -9.08, *p* < .001, SE Cohen’s d = 1.14). An ANOVA explored the effect of the different conditions (training, reversal, escape) on proportion of choice for the discriminative alternative during the last 5 blocks of trials of each condition, indicating no effect of condition (F(2, 10) = 0.08, *p* = .93; partial η^2^ = 0.015), session (F(4, 20) = 1.89, *p* = .15; partial η^2^ = 0.27) nor its interaction (F(8, 40) = 0.90, *p* = .53; partial η^2^ = 0.15).

In regard to aspects of discrimination, Fig. 3 panel c shows that beginning in the fifth block of trials, the latency to enter the door associated with the negative outcome was notably larger than the latency to cross the doors associated with the positive, ND1 and ND2 outcomes. An ANOVA evaluated the effect of type of outcome and session on latency during the last five blocks of trials of the training condition, indicating a significant effect of type of outcome (F(1, 5.02) = 5.72, *p* < .05; partial η^2^ = 0.64; Greenhouse-Geisser correction was implemented), while the effects of block of trials (F(4, 20) = 1.30, *p* = .30; partial η^2^ = 0.21) and its interaction with type of outcome were not (F(12, 60) = 1.60, *p* = .12; partial η^2^ = 0.24). Scheffé post-hoc analysis revealed that the latency to cross the door of the negative outcome was larger than that to cross the door of the positive outcome, and that the latencies to cross the doors associated with the ND1 and ND2 were equivalent. Figure 3 panel d shows the group mean of the individual medians of the time taken to traverse each of the tunnels associated with the different outcomes. The effect of type of outcome was significant (F(1.28, 6.42) = 7.11, *p* < .05; partial η^2^ = 0.59), while the effects of block of trials (F(4, 20) = 0.22, *p* = .92; partial η^2^ = 0.04) and its interaction with type of outcome were not (F(12, 60) = 0.41, *p* = .95; partial η^2^ = 0.08). Post-hoc analyses indicated a significant difference between the traversing time of the negative and positive tunnels, but no significant difference between the traversing time of the ND1 and ND2 outcomes. Data obtained during the last five blocks of trials during the reversal condition regarding the above-described variables were analyzed in the same way than data from the training condition. Without exception, both significant and non-significant results were replicated. Online resource 2 shows the statistical details of those analyses.

Figure 3 Panel e shows the proportion of trials with escape for each type of outcome. It can be observed that since the first block of trials, a high proportion of escape (0.61) was observed for the negative outcome, and remained above 0.84 for the entire condition. It is Important to highlight that the proportion of trials with escape for the other three types of outcomes remained in zero for the entire duration of the condition, demonstrating that subjects were able to discriminate between the negative outcome and the three other outcomes.

### Discussion

In this experiment we replicated the preference for the non-discriminative alternative that was found in experiment 1 of the present report. This was indicated by data derived from both forced trials and free-choice trials. In regard to the former, shorter latencies were found for the non-discriminative alternative than for the discriminative alternative during the final blocks of trials of each condition, and in regard to the latter, the proportion of choice for the discriminative alternative was consistently low at the end of each of the conditions. This strong preference for the optimal alternative occurred with different probabilities for each of the alternatives (0.2 vs. 0.5) than for experiment 1 (0.5 vs. 0.75), with longer tunnels (1.5 m) than in experiment 1 (0.5 m), and in a closed economy condition. The consistency of the preference for the optimal alternative despite these procedural differences suggests the broad generality of our main finding.

During training and reversal conditions we also replicated the high level of discrimination between the positive and the negative outcomes of the discriminative alternative: both the latency to enter the door of the negative outcome and the traversing time of the negative tunnel were higher than those related to the positive outcome. Both results validate the data on preference discussed above, as they are evidence that the preference for the optimal alternative is not an artifact of generalization between the positive and the negative outcomes, as has been suggested as an explanation of the differences between rats and pigeons in the suboptimal choice procedure (Zentall et al., 2019b).

An extra piece of evidence about discrimination can be obtained from the “escape allowance” condition: since the first block of trials with opportunity to escape from the outcomes found, subjects presented a high proportion of escape from the negative outcome, which prevailed during the rest of that condition. In contrast, the proportion of escape from the positive outcome was zero during all blocks of trials.

Finally, the closed economy regime did not generate an obvious difference in preference respect the open economy regime of experiment 1. The proportion of choice for the discriminative alternative was equally low for both experiments. In regard to latencies to enter the different alternatives, we found that absolute values were longer in closed economy than in open, but despite that, in both experiments the latencies to enter the non-discriminative alternative were consistently shorter than those to enter the discriminative alternative.

## General discussion

The results of the present experiments concur in signaling that rats’ optimality found in previous studies with operant chambers also occurred with the use of a procedure that presented several differences respect the operant-chambers procedure: (a) both the alternatives (discriminative and non-discriminative) and its associated outcomes (positive, negative, ND1 and ND2) were defined exclusively in spatial terms, (b) the response for choosing between alternatives was locomotion, unlike the majority of previous studies in which lever-pressing or nose-poking were required, and (c) when the outcomes were found, locomotion was required to get access to the final consequence either reinforcer delivery or its absence. Derived from these procedural differences, the discrimination indexes were necessarily distinct to the traditional analysis based on the difference in number of lever presses or entrances to the feeder in the presence of the positive and negative stimuli. Instead of them, we analyzed the latency to enter the doors associated with these outcomes, the traversing time of the tunnels for reaching the goal box, and the proportion of escape when the positive and the negative outcomes were found. These three conceptually-distinct indexes of discrimination coincide in indicating that rats readily discriminated between the positive and the negative outcomes of the discriminative alternative, supporting the validity of the adaptation of the suboptimal choice procedure to the apparatus employed in the present experiments.

In addition, a closed economy was simulated by providing food exclusively during the experimental sessions, and by not limiting the number of reinforcers that could be obtained during them, conditions that satisfy the definition of a closed economy (Hursh 1980). For achieving the goal of not providing extra food, long sessions were required. Under these circumstances, we found no relevant differences between open (exp. 1) and closed (exp. 2) economies. Although it has been reported that the type of economy employed has large effects on the determination of the value of a reinforcer (for a review, see Kearns 2019), and also on the relationship between a given schedule of reinforcement and the response rate that it maintains (Posadas-Sánchez and Killeen 2005), a series of experiments on choice behavior reported no differential effects of open and closed economies (Fantino and Abarca 1985). In the context of the experiments reported here, the use of a closed economy had the only purpose of performing a more suitable simulation of natural foraging.

Our procedure bears some resemblances with simulations of natural foraging that have been widely employed for evaluating in operant chambers predictions of optimal-diet models (Fantino and Abarca 1985; Hanson 1987; Lea 1979). The prototypical procedure, the “successive-encounters procedure” (Lea 1979), has as main characteristics the simulation of the different phases of a foraging episode, i.e., search, choice, handling and consumption. After satisfying a reinforcement schedule that represents the search phase, normally a fixed-interval (Lea 1979) or a variable-interval schedule (Abarca and Fantino 1982), the choice phase is presented in which either of two “preys” is found. In this stage, organisms have the opportunity of either accepting or rejecting the outcome found; if accepted, the handling stage is initiated and the reinforcer is provided upon its completion; if rejected, a new search is initiated. After reinforcer consumption, a new search begins in which the same or the alternative prey can be found. In this way, organisms never choose directly between the two outcomes, but only choose to accept or reject each of them in a given moment. In our procedure the choice phase and the handling phase were clearly separated, the outcomes were presented sequentially rather than simultaneously (in most sessions of experiment 1) and subjects had the opportunity of rejecting the outcome that was presented (third condition of experiment 2). These characteristics, joined to Timberlake’s discussion about how rats’ performance in mazes benefits from the remarkable similarity between mazes and rats’ natural environment, strengthen the analogy with natural foraging (Timberlake 2002). Simulating foraging has proven to be an elusive task (Fantino and Abarca 1985), and we do not regard the procedure of the present experiments as a thorough simulation of natural foraging. Nevertheless, we consider that the employed procedure adequately faced the subjects with several conditions that have been regarded as useful when evaluating predictions from optimal-diet models (in particular those related to item selection), thus representing an ecologically-valid procedure.

In the context of research on suboptimal choice, the possibility of escaping from the outcomes represents a novel and relevant aspect of the procedure. Besides providing an additional measure of discrimination, the possibility of escaping models the natural foraging situation that has been proposed as the evolutionary basis of pigeons’ and starlings’ preference for the discriminative alternative (Vasconcelos et al. 2015). The implementation of the possibility of escape was successful, indicated both by the readiness with which the subjects learned the escape response, and by the high probability with which it was observed. For comparison with related research, when in a suboptimal choice procedure with 10-s long terminal links and 10-s long inter-trial Intervals (ITI), pigeons were given the opportunity of escaping from the outcomes by pecking an “escape key” (Fortes et al. 2017), the probability of escape was 0.26 (after forced trials for escaping) and increased to 0.8 when ITIs were decreased to 1s; with 20-s TLs, the escape proportion was about 0.52, i.e., notably smaller than the level observed in the present experiment. In regard to research on rats, the only study that has allowed an escape response was not successful in generating it, as none of the rats showed a consistent escape response (Roper and Baldwin 2004). Noteworthy to mention is that subjects in the present report did not receive any training for escaping; the only manipulation that was performed was to open the door that led subjects to the beginning of the apparatus, where the open starting door signaled the availability of a new trial. In this context, it is possible that the readiness with which rats emitted the escape response is related to the use of an escape-response topography (running away from the negative outcome) similar to that employed in natural foraging to escape from the signals of no reinforcement, suggesting that our procedure adequately modeled the natural situation in which animals abandon pursuing a prey that won’t be captured, in search of other food opportunities. Related to this, the rapid learning of the escape response found in the present experiment, together with its specificity -animals only escaped from the negative outcome- suggest a strong component of aversiveness of the negative outcome, which could be the mechanism that drives the preference for the alternative without such kind of aversive outcomes, i.e., the non-discriminative and optimal (Trujano et al. 2016). Not consistent with this view is the fact that even though the probability of escaping from the negative outcome approached 1, there was not an increase in preference for the discriminative alternative. It is possible that this was due to the fact that the time (and effort) employed in escaping were not sufficiently shorter than the combined costs of traversing the negative tunnel and the return tunnel, so that the costs associated with the discriminative alternative were not lowered enough for an increase in preference to occur. Further experiments could test this idea by employing longer outcome tunnels and/or diminishing the costs associated with escaping.

The high probability of escaping could contribute to the current debate about the role that the negative stimulus has on the determination of rats’ and pigeons’ preference. Laude et al. (2014) reported that in stable state the negative stimulus did not have conditioned-inhibition properties, which was regarded as the base for the development of pigeons’ suboptimal preference. This account is consistent with the results reported by Trujano et al. (2016), in which the strong conditioned inhibition that was found in rats was assumed to explain their optimal preferences. However, subsequent research with pigeons (González and Blaisdell 2021) reported not only that suboptimal preferences were found despite the negative stimulus had conditioned-inhibition properties, but that the strength of the conditioned inhibition correlated with suboptimal preferences. This analysis suggests that the conditioned-inhibition properties are not enough for explaining the development of suboptimal preferences and suggests the need of analyzing the impact of other variables. One of these variables could be the aversiveness of the negative stimulus, which could affect suboptimal preferences by punishing the choice of the discriminative alternative. If the aversive properties of the negative stimulus are differentially developed in pigeons and rats, then the differential development of suboptimal preferences would be understood. The high probability of escaping from the negative stimulus by rats of the present study in comparison with the lower probability of escaping by pigeons in the study by Fortes et al. (2017) is consistent with this view.

In the conditions in which no escape was possible, subjects also preferred the optimal alternative despite differences in the parameters of some variables that have been considered relevant in the study of suboptimal choice. First, rats preferred the optimal alternative with two distinct combinations of probabilities for the discriminative and the non-discriminative alternative: 0.50 vs. 0.75 (a ratio of 1:1.5) in experiment 1, and 0.20 vs. 0.5 (a ratio of 1:2.5) in experiment 2. Although the relative probability of reinforcement associated with the discriminative alternative is poorer with the second combination than with the first, pigeons show a higher preference for the discriminative alternative with the second pair of probabilities (Gipson et al. 2009; Stagner and Zentall 2010), a counterintuitive result that is consistent with both, the contrast account of suboptimal choice (Zentall 2016; Zentall et al. 2019a), and the SiGN Model (Dunn et al. 2024). In particular, for the present experiments the SiGN model predicts a 0.27 proportion of choice for the discriminative alternative with the 0.5 vs. 0.75 combination of probabilities and 0.65-s TL’s length (experiment 1) and a 0.36 proportion with the 0.2 vs. 0.5 combination of probabilities and 1.5-s TL’s length (experiment 2). The Sigma-Delta model (González et al. 2020), in contrast to these previous models, predicts a higher preference for the discriminative alternative for the 0.50 vs. 0.75 combination of probabilities (if Deltas and the sensitivity parameters β and c are kept constant).

Experiments 1 and 2 also differed in the length of the procurement tunnels associated with the different type of outcomes, 50 cm in experiment 1 vs. 150 cm in experiment 2. The rationale for this manipulation was to follow up the report of the emergence of suboptimal preferences in rats when the terminal links’ length was increased (Cunningham and Shahan 2019). Given that in the present report no difference between experiments was found in the proportion of choice for the discriminative alternative, we conclude that this variable did not have an effect on increasing suboptimal preferences. Although it is possible that the longer tunnels of experiment 2 still represent terminal links shorter than the length needed for suboptimal preferences to emerge, as the traversing times were normally shorter than 2 s, it should be considered that the subjects not only spent time, but also effort, which undoubtedly increased the costs associated with food-procurement during the terminal links. Even if only the delay to outcome and not the effort invested were considered, a slight preference for information was found in a study with humans with terminal links of 1 s (Iigaya et al. 2020) and a strong preference for information was found with Rhesus macaque monkeys (*Macaca mulatta*) with terminal links of 2.25 s (Bromberg-Martin and Hikosaka 2009). This comparison suggests that the optimality that we found does not depend on parametric issues related to the terminal-links’ length. Consistent with this view, preference for the optimal alternative has been found in studies with rats performed in operant chambers with terminal links as long as 50 s (Alba et al. 2021), and the preference for the discriminative alternative with 50-s long TLs reported by Cunningham and Shahan (2019) was not replicated in a later experiment (Cunningham and Shahan 2020). Nevertheless, studies with longer terminal links than those employed here are still needed, especially because of the relevance that different quantitative models of suboptimal choice like the SiGN Model (Dunn et al. 2024), the temporal-information theoretic model (Cunningham and Shahan 2018), and the anticipatory utility model (Iigaya et al. 2016) ascribe to temporal variables (For a review of the relevance of temporal context on suboptimal choice, see Cunningham and Shahan 2018; Dunn et al. 2024). In particular, although for the terminal links’ time obtained in our experiments, the SiGN model predicts preference for the optimal alternative (Pisklak 2024), suboptimal behavior is predicted for terminal links as short as 4 s, a prediction that has not been confirmed in studies with rats performed in operant chambers, but that is warranted to assess employing the present procedure. It would also be ideal that those experiments control the time spent in the procurement tunnels, because our current design allows variability in the terminal-links time, as it depends on each subject’s running speed. This modification could be achieved by employing additional doors that restrict the exit from the procurement tunnels until the criteria time has elapsed.

Setting aside the issue of the relevance of terminal link’s length on rats’ preferences, the majority of studies performed with the 0.2 vs. 0.5 arrangement of probabilities of reinforcement for the discriminative and non-discriminative alternative and 10-s long terminal links have found optimal preferences (Alba et al. 2018, 2021; Cunningham and Shahan 2019, 2020; Ojeda et al. 2018; Trujano and Orduña 2015), so that the study that found suboptimal preferences under these parameters seems to be the exception (Chow et al. 2017). Rats’ preferences, then, are at odds with pigeons’ and starlings’ preferences under the same set of parameters, as they show strong and consistent preferences for the suboptimal alternative. Given the clear suboptimality of pigeons’ behavior, the procedure was regarded as a model of human maladaptive behavior (Zentall 2014). This idea was strengthened by the report of suboptimality in a sample of human gamblers (Molet et al. 2012). Were the same mechanism underly pigeons’ and humans’ suboptimal behavior, wide generality among species should be expected, including rats. Given that studies previous to the first report of rats’ optimality (Trujano and Orduña 2015) coincided in finding suboptimal behavior in species as different as pigeons and humans, rats’ optimality was regarded as an anomaly, and the reasons for that anomaly were looked for (Zentall et al., 2019b). Currently, together with the replication in different laboratories of rats’ optimality, new evidence about human optimality in the suboptimal choice procedure has been found. Recently, McDevitt et al. (2019), for example, replicated the Molet et al’s study (2012) in a 60-participants, non-gamblers sample, finding that the proportion of choice for the suboptimal alternative was 0.26, i.e., very close to what has been found in studies performed with rats in operant chambers. Other studies coincide in the demonstration of humans’ optimality (for a review, see Bodily and Bodily 2024). Furthermore, research performed with procedures different than suboptimal choice have shown that human participants do value advanced information on the results of probabilistic reinforcers, but when that information is costly (as in the suboptimal choice procedure), humans prefer the no-information alternative (Brydevall et al. 2018). Conjoint with these reports of optimality in rats and humans, recent research has focused on finding the determinants of pigeons’ suboptimal behavior. It has been demonstrated, for example that pigeons’ suboptimal behavior of pigeons is restricted to choice situations in which the initial links are very short (McDevitt et al. 2024), or procedures in which the choice response is key-pecking (González-Torres et al. 2020). This evidence suggests that rats’ optimality in the “suboptimal-choice procedure” is a relevant part of converging evidence of optimality in species different than pigeons or starlings.

In summary, we replicated in both experiments the preference of rats for the optimal alternative. The appropriate levels of discrimination that were found discard the possibility that the optimal preference was an artifact of the experimental design. Given the novel procedure employed in the present experiments, such findings suggest that rats’ optimality has a high degree of generality. The incorporation of the present findings to the analysis of the performance of different species in the suboptimal-choice procedure would contribute to the ultimate goal of understanding the variables that determine suboptimal choice in some species, which in turn could provide tools for treating problems of maladaptive behavior in people vulnerable to the influence of those variables.

## Electronic supplementary material

Below is the link to the electronic supplementary material.

Supplementary Material 1

Supplementary Material 2

Supplementary Material 3

Supplementary Material 4

Supplementary Material 5

## Data Availability

Data will be available upon request.
