# Supplementary material for: Rats’ performance in a suboptimal choice procedure implemented in a natural-foraging analogue
Source: Anim Cogn. 2024 Nov 1;27(1):72. doi: 10.1007/s10071-024-01913-2 (PMC11530512; doi:10.1007/s10071-024-01913-2)
Supplement: Supplementary file 1 — Supplementary Material 1 [file 10071_2024_1913_MOESM1_ESM.pdf]

Table 1. Summary of the statistical results related to the variables derived from experiment 1. A repeated measures ANOVA was performed for each variable in each condition (training, reversal). The symbol * denotes that the assumption of sphericity was violated, and that the Greenhouse-Geisser correction was implemented.

	TRAINING	REVERSAL
LATENCY TO DISCRIMINATIVE AND NON-DISCRIMINATIVE ALTERNATIVES	ALTERNATIVE: F(1, 7)=181.81, p<.0001; partial η2 = .962	ALTERNATIVE: F(1, 7)=395.95, p<.0001; partial η2 =.98
	SESSIONS: F(4, 28)=2.64, p=.055; partial η2 = .27	*SESSIONS: F(2.13, 14.90)=10.31, p<.001; partial η2 =.59
	ALTERNATIVE X SESSIONS: F(4, 28)=.96, p=.44; partial η2 = .12	*ALTERNATIVE X SESSIONS: F(1.99, 13.98)=4.94, p<.05; partial η2 =.41
LATENCY TO OUTCOMES: ND1, ND2, POSITIVE, NEGATIVE	*OUTCOME: F(1, 7)=28.68, p<.001, partial η2 =.80	*OUTCOME: F(1.01, 7.06)=31.60, p<.001; partial η2 =.82
	SESSIONS: F(4, 28)=.99, p=.43, partial η2 =.12	*SESSIONS: F(1.45, 10.14)=.59, p=.67; partial η2 =.08
	OUTCOME X SESSIONS: F(12, 84)=1.01, p=.45, partial η2 =.12	OUTCOME X SESSIONS: F(12, 84)=.55, p=.88; partial η2 =.07
TRAVERSING TIME OF OUTCOMES ND1, ND2, POSITIVE, NEGATIVE	*OUTCOME: F(1.02, 7.13)=10.32, p<.001; partial η2 =.60	*OUTCOME: F(1.05, 7.01)=4.43, p=.07; partial η2 =.39
	SESSIONS: F(4, 28)=.53, p=.71; partial η2 =.07	SESSIONS: F(4, 28)=1.56, p=.21; partial η2 =.18
	OUTCOME X SESSIONS: F(12, 84)=.45, p=.94; partial η2 =.06	OUTCOME X SESSIONS: F(12, 84)=1.12, p=.35; partial η2 =.14
PROPORTION OF CHOICE FOR THE DISCRIMINATIVE ALTERNATIVE		
	t (7) = -31.71, p < .001, SE Cohen's d = 2.82	t (7) = -36.69, p < .001, SE Cohen's d = 3.26
