# Supplementary material for: Rats’ performance in a suboptimal choice procedure implemented in a natural-foraging analogue
Source: Anim Cogn. 2024 Nov 1;27(1):72. doi: 10.1007/s10071-024-01913-2 (PMC11530512; doi:10.1007/s10071-024-01913-2)
Supplement: Supplementary file 2 — Supplementary Material 2 [file 10071_2024_1913_MOESM2_ESM.pdf]

TABLE 2 Summary of the statistical results related to the variables derived from experiment 2. A repeated measures ANOVA was performed for each variable in each condition (training, reversal, escape). The symbol * denotes that the assumption of sphericity was violated and that the Greenhouse-Geisser correction was implemented.

	TRAINING	REVERSAL	ESCAPE
LATENCY TO DISCRIMINATIVE AND NON-DISCRIMINATIVE ALTERNATIVES	ALTERNATIVE: F(1, 5)=35.67, p<.01; partial η2 =.88	ALTERNATIVE: F(1, 5)=24.46, p<.01; partial η2 =.83	ALTERNATIVE: F(1, 5)=7.32, p<.05; partial η2 =.59
	BLOCK OF TRIALS: F(4, 20)=2.13, p=.11; partial η2 =.30	BLOCK OF TRIALS: F(4, 20)=1.33, p=.29; partial η2 =.21	BLOCK OF TRIALS: F(4, 20)=.80, p=.54; partial η2 =.14
	* ALTERNATIVE X BLOCK OF TRIALS: F(1.52, 7.61)=.14, p=.96; partial η2 =.03	* ALTERNATIVE X BLOCK OF TRIALS: F(1.35, 6.75)=1.18, p=.35; partial η2 =.19	ALTERNATIVE X BLOCK OF TRIALS: F(1.19, 5.94)=1.17, p=.33; partial η2 =.19
LATENCY TO OUTCOMES: ND1, ND2, POSITIVE, NEGATIVE	* OUTCOME: F(1, 5.02)=5.72, p<.05; partial η2 =.64	* OUTCOME: F(1.06, 5.3)=12.51, p<.05; partial η2 =.72	
	BLOCK OF TRIALS: F(4, 20)=1.30, p=.30; partial η2 =.21	*BLOCK OF TRIALS: F(1.92, 9.59)=1.04, p=.39; partial η2 =.17	
	OUTCOME X BLOCK OF TRIALS: F(12, 60)=1.60, p=.12; partial η2 =.24	OUTCOME X BLOCK OF TRIALS: F(12, 60)=.86, p=.59; partial η2 =.15	
TRAVERSING TIME OF OUTCOMES ND1, ND2, POSITIVE, NEGATIVE	* OUTCOME: F(1.28, 6.42)=7.11, p<.05; partial η2 =.59	* OUTCOME: F(1.10, 5.5)=7.65, p<.05; partial η2 =.60	
	BLOCK OF TRIALS: F(4, 20)=.22, p=.92; partial η2 =.04	BLOCK OF TRIALS: F(4, 20)=1.23, p=.33; partial η2 =.20	
	OUTCOME X BLOCK OF TRIALS: F(12, 60)=.41, p=.95; partial η2 =.08	OUTCOME X BLOCK OF TRIALS: F(12, 60)=1.84, p=.06; partial η2 =.27	
PROPORTION OF CHOICE FOR THE DISCRIMINATIVE ALTERNATIVE			
	t (5) = -9.08, p < .001, SE Cohen's d = 1.14	t () = -18.28, p < .001, SE Cohen's d = 2.19	t (7) = -16.12, p < .001, SE Cohen's d = 1.94
