# Supplementary material for: Rats’ performance in a suboptimal choice procedure implemented in a natural-foraging analogue
Source: Anim Cogn. 2024 Nov 1;27(1):72. doi: 10.1007/s10071-024-01913-2 (PMC11530512; doi:10.1007/s10071-024-01913-2)
Supplement: Supplementary file 4 — Supplementary Material 4 [file 10071_2024_1913_MOESM4_ESM.pdf]

Supplementary Online Materials

Individual data from experiment 1

FIG S1a. Experiment 1. Individual median latencies to discriminative and non-discriminative alternatives

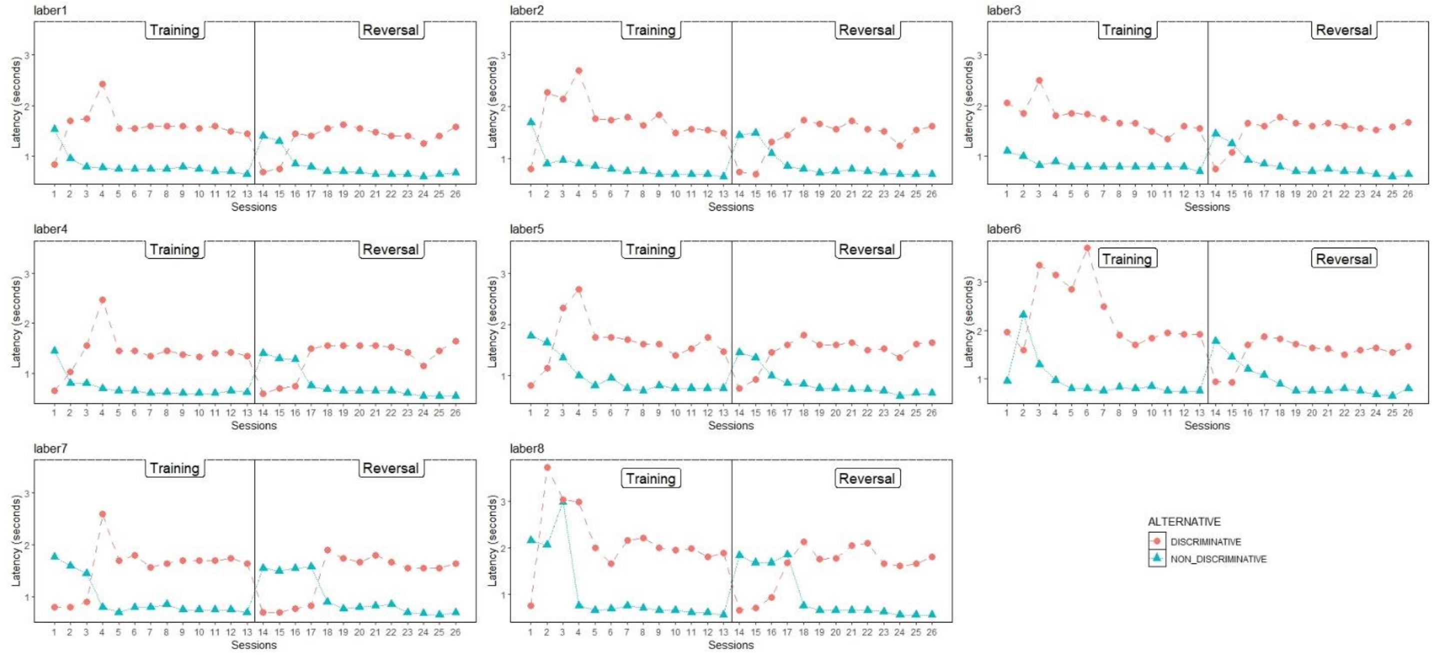

Fig. S1a Experiment 1. Individual median latencies for entering the doors associated with the discriminative and non-discriminative alternatives. The vertical line in each panel divides data obtained during the original training condition from those obtained during the reversal phase

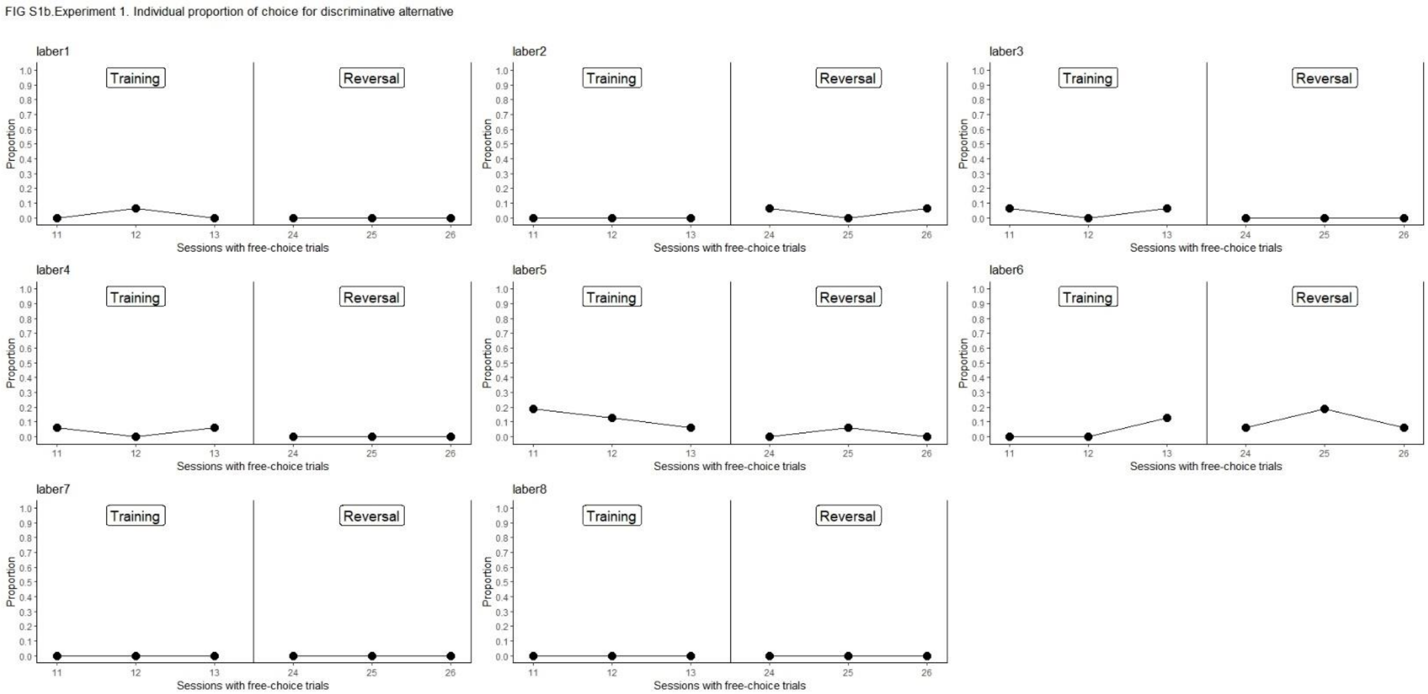

Fig. S1b Experiment 1. Individual proportion of choice for the discriminative alternative during each session with choice trials. The vertical line in each panel divides data obtained during the original training condition from those obtained during the reversal phase

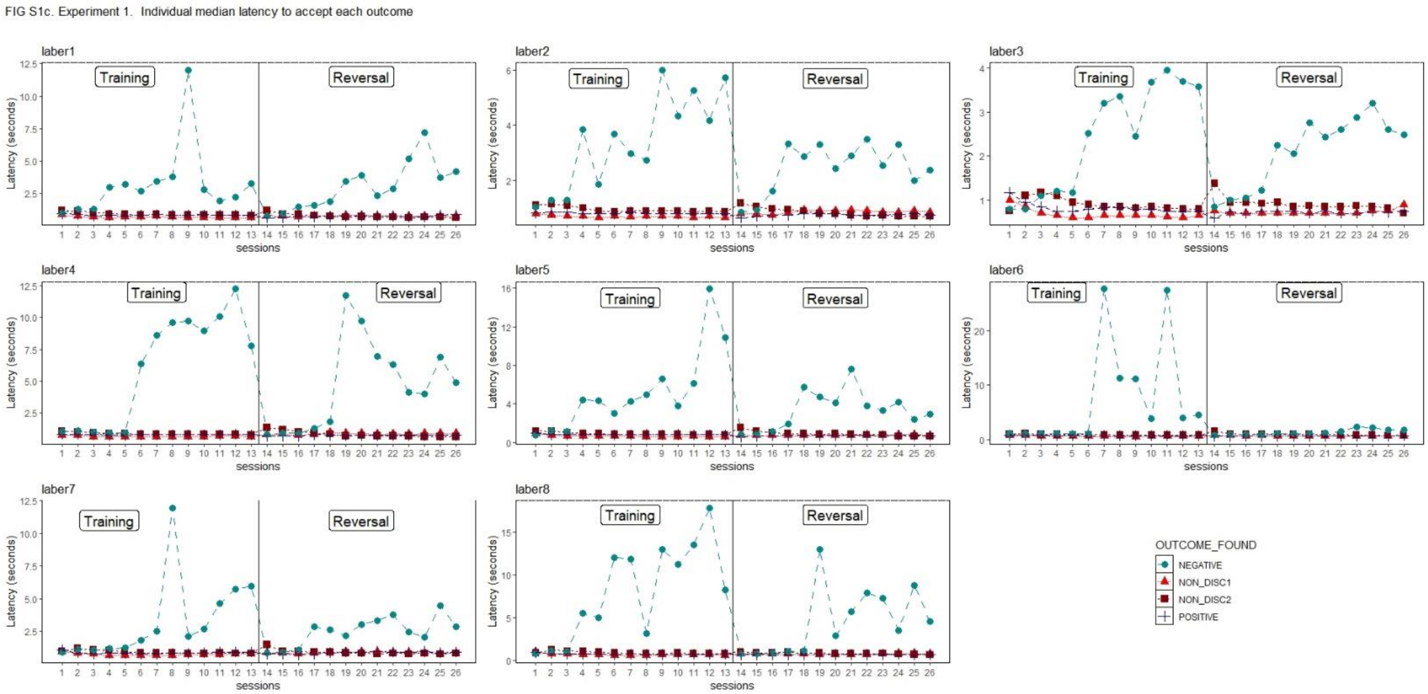

Fig. S1c Experiment 1. Individual median latencies for entering the door associated with each of the four possible outcomes (positive, negative, ND1 and ND2). The vertical line in each panel divides data obtained during the original training condition from those obtained during the reversal phase

Fig. S1d. Experiment 1. Individual traversing time for each outcome

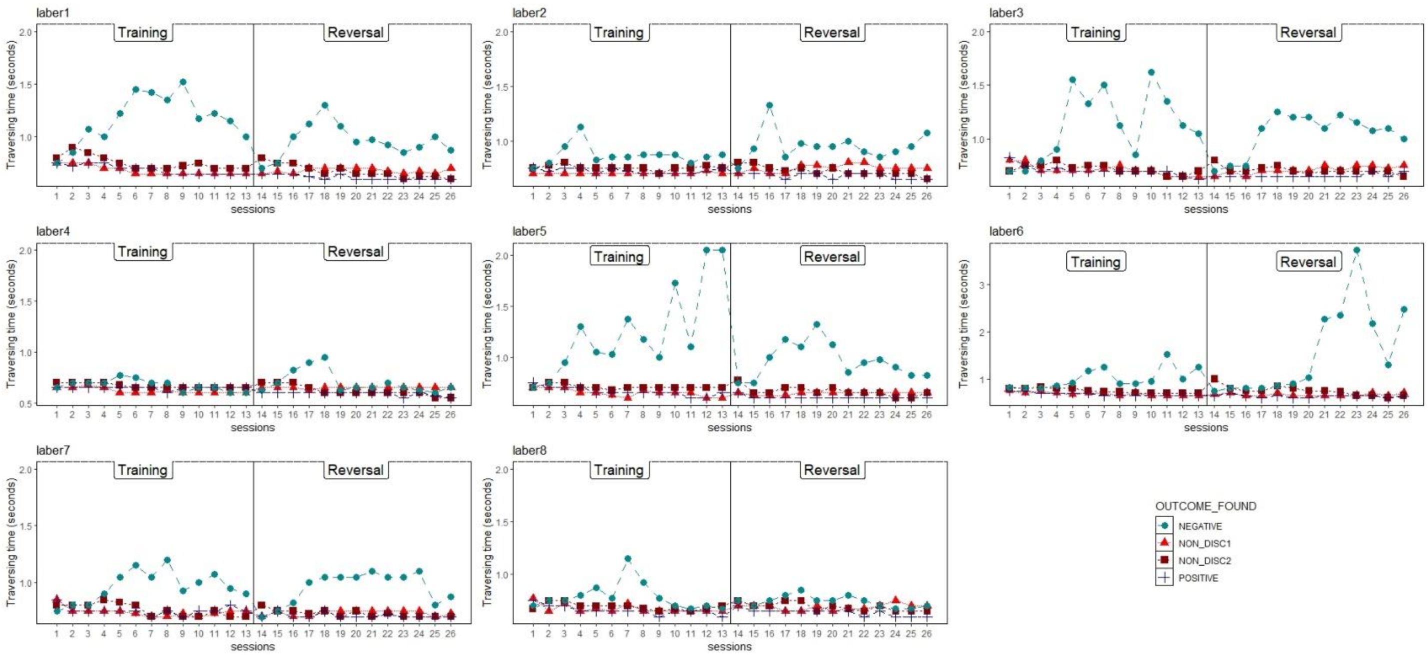

Fig. S1d Experiment 1. Individual median of the traversing time of the tunnel associated with each of the possible outcomes (positive, negative, ND1 and ND2). The vertical line in each panel divides data obtained during the original training condition from those obtained during the reversal phase
