# Supplementary material for: Rats’ performance in a suboptimal choice procedure implemented in a natural-foraging analogue
Source: Anim Cogn. 2024 Nov 1;27(1):72. doi: 10.1007/s10071-024-01913-2 (PMC11530512; doi:10.1007/s10071-024-01913-2)
Supplement: Supplementary file 5 — Supplementary Material 5 [file 10071_2024_1913_MOESM5_ESM.pdf]

Supplementary Online Materials

Individual data from experiment 2

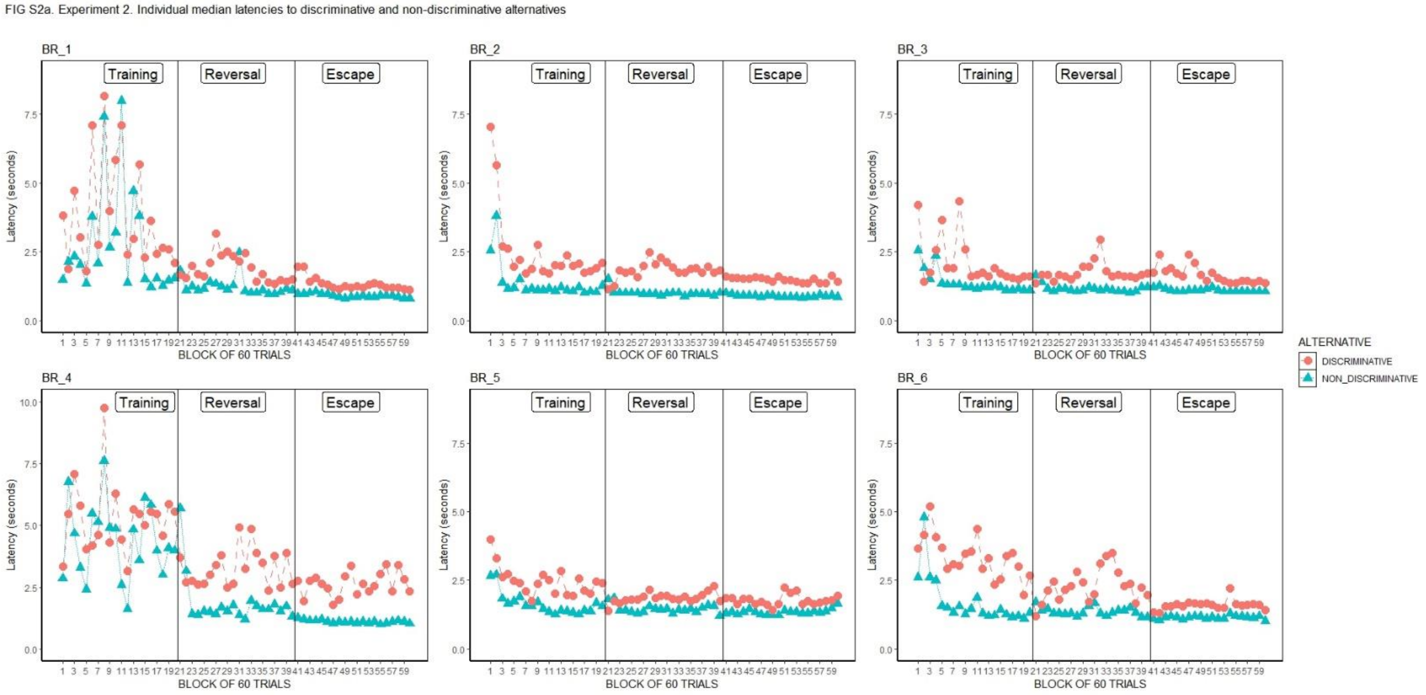

Fig. S2a Experiment 2. Individual median latencies for entering the doors associated with the discriminative and non-discriminative alternatives across blocks of 60 trials during training, reversal, and escape phases. The vertical lines in each panel divides data obtained during the original training phase from those obtained during the reversal and escape phases

FIG S2b.Experiment 2. Individual proportion of choice for discriminative alternative

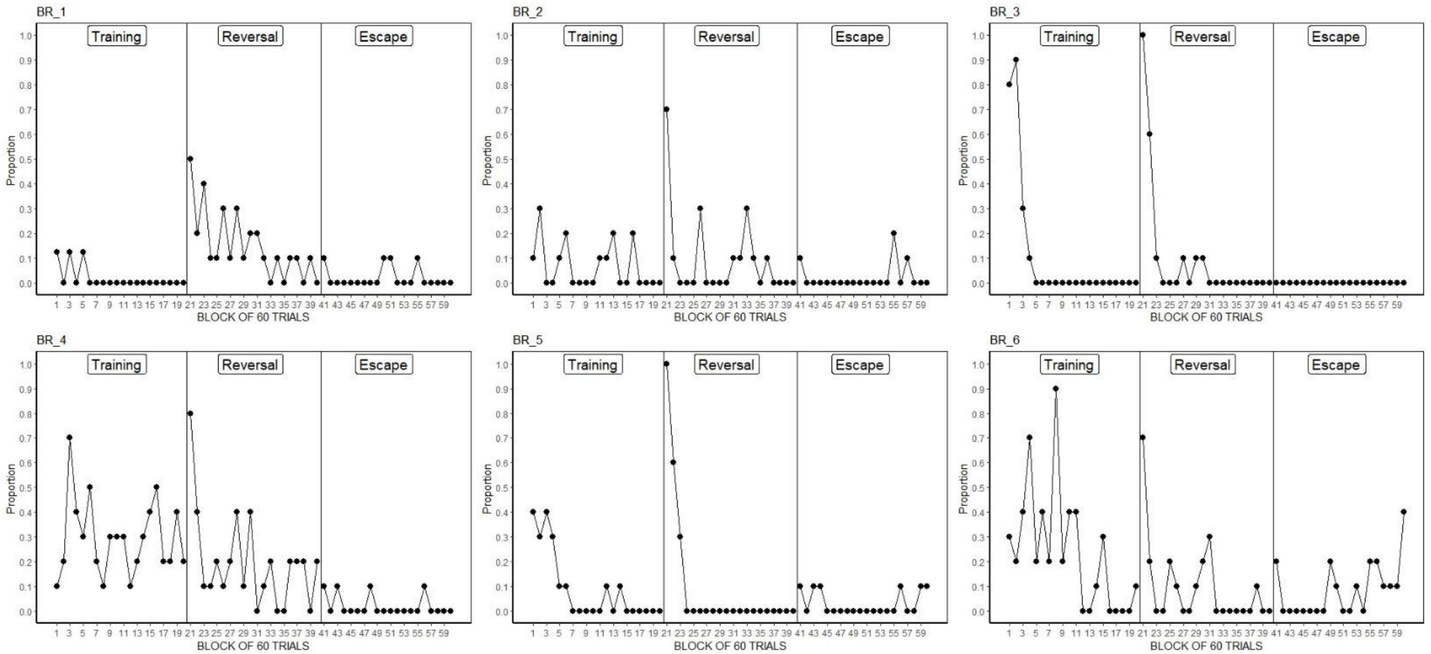

Fig. S2b Experiment 2. Individual proportion of choice for the discriminative alternative during each block of 60 trials during training, reversal, and escape phases. The vertical lines in each panel divides data obtained during the original training phase from those obtained during the reversal and escape phases

FIG S2c. Experiment 2. Individual median latency to accept each outcome

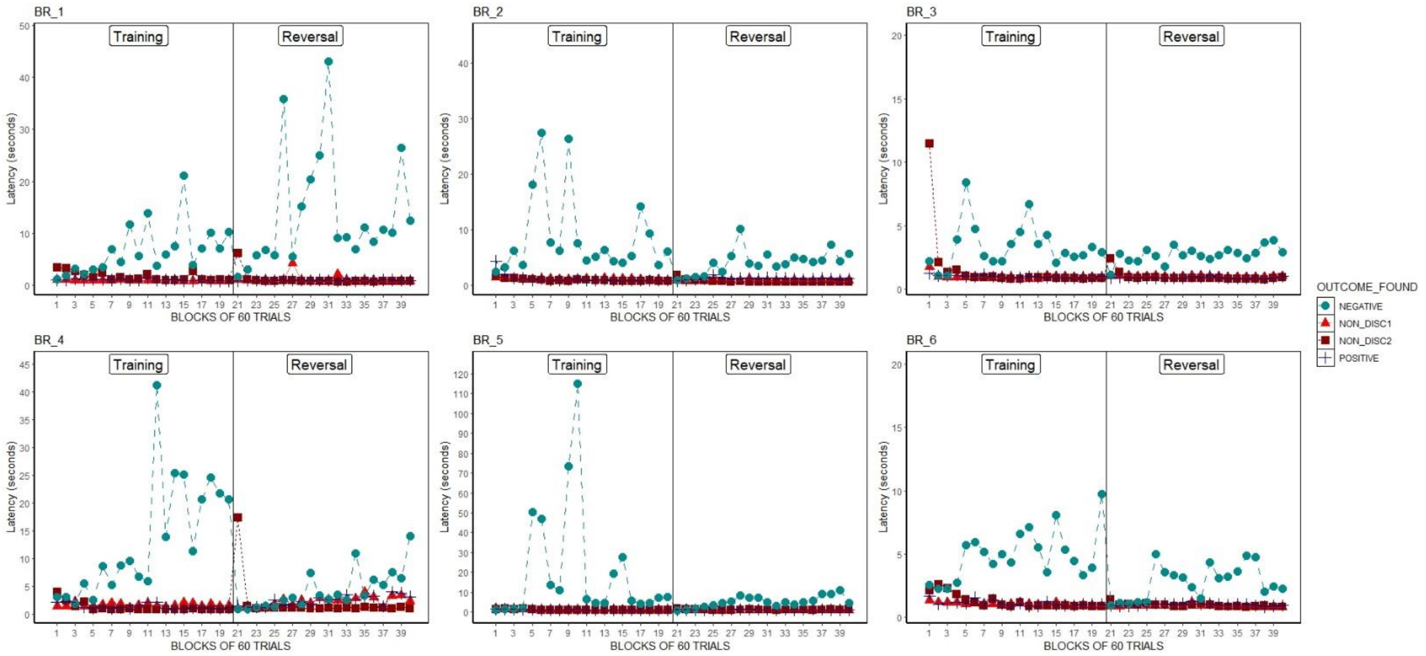

Fig. S2c Experiment 2. Individual median latencies for entering the door associated with each of the four possible outcomes (positive, negative, ND1 and ND2) across blocks of 60 trials during training and reversal phases. The vertical line in each panel divides data obtained during the original training phase from those obtained during the reversal phase

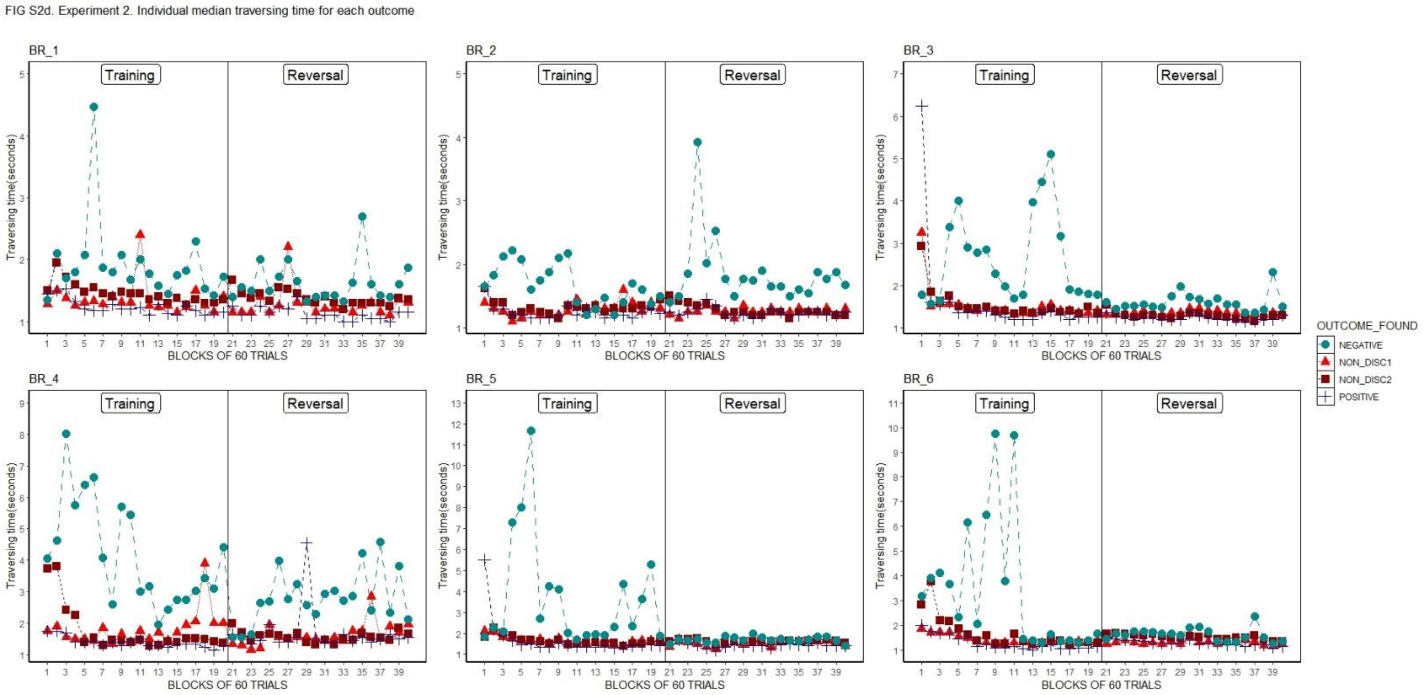

Fig. S2d Experiment 2. Individual median of the traversing time of the tunnel associated with each of the possible outcomes (positive, negative, ND1 and ND2) across blocks of 60 trials during training and reversal phases. The vertical line in each panel divides data obtained during the original training phase from those obtained during the reversal phase

FIG S2e. Experiment 2. Individual proportion of escape

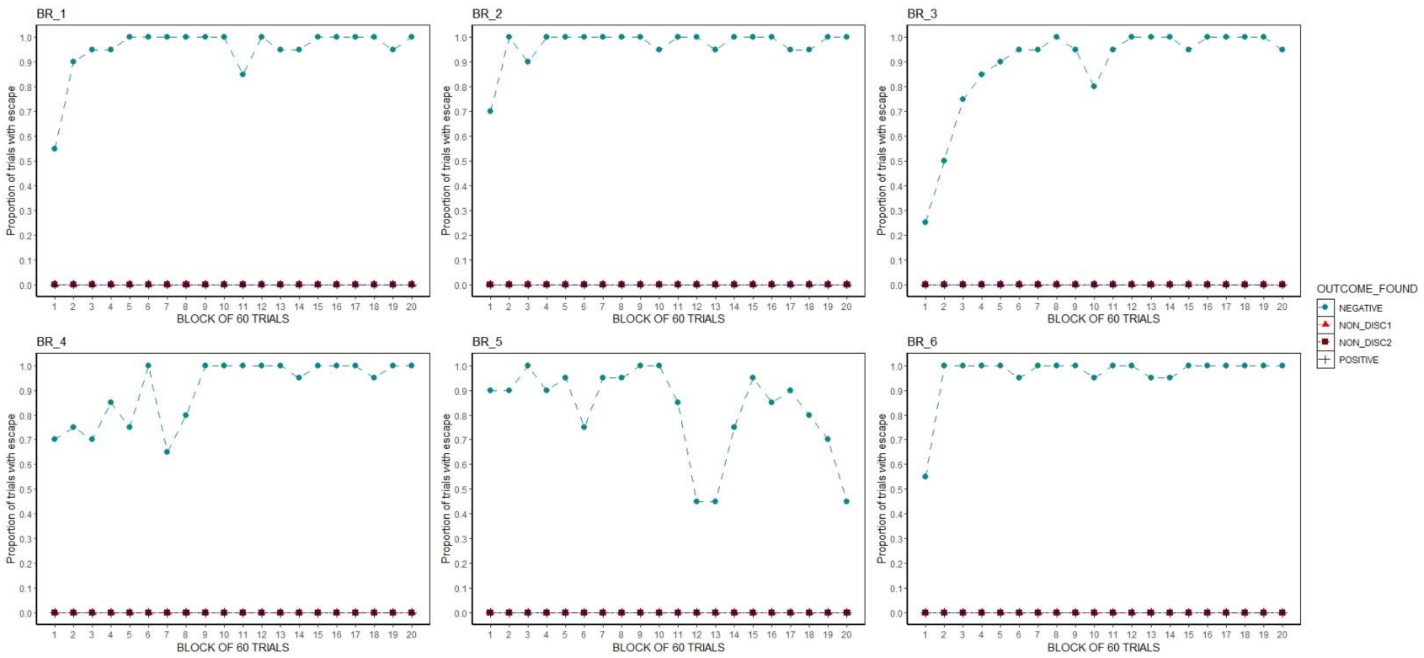

Fig. S2e Experiment 2. Individual proportion of trials with escape for each of the four possible outcomes found (positive, negative, ND1 and ND2)
